# Sarcopenia: investigation of metabolic changes and its associated mechanisms

**DOI:** 10.1186/s13395-022-00312-w

**Published:** 2023-01-19

**Authors:** Jair Marques, Engy Shokry, Olaf Uhl, Lisa Baber, Fabian Hofmeister, Stefanie Jarmusch, Martin Bidlingmaier, Uta Ferrari, Berthold Koletzko, Michael Drey

**Affiliations:** 1 Department of Paediatrics, LMU - Ludwig-Maximilians-Universität Munich, Division of Metabolic and Nutritional Medicine, Dr. von Hauner Children’s Hospital, University Hospital, LMU Munich, Lindwurmstr, 4, D-80337 Munich, Germany; 2 Department of Medicine IV, University Hospital, LMU Munich, Munich, Germany

**Keywords:** Sarcopenia, Metabolomics, Energy metabolism, Mitochondrial metabolism, Fatty acid metabolism, Lipid metabolism, Fatty acid oxidation

## Abstract

**Background:**

Sarcopenia is one of the most predominant musculoskeletal diseases of the elderly, defined as age-related progressive and generalized loss of muscle mass with a simultaneous reduction in muscle strength and/or function. Using metabolomics, we aimed to examine the association between sarcopenia and the plasma metabolic profile of sarcopenic patients, measured using a targeted HPLC-MS/MS platform.

**Methods:**

Plasma samples from 22 (17 men) hip fracture patients undergoing surgery (8 sarcopenic, age 81.4+6.3, and 14 non-sarcopenic, age 78.4±8.1) were analyzed. *T* test, fold change, orthogonal partial least squares discriminant analysis, and sparse partial least squares discriminant analysis were used for mining significant features. Metabolite set enrichment analysis and mediation analysis by PLSSEM were thereafter performed.

**Results:**

Using a univariate analysis for sarcopenia *z* score, the amino acid citrulline was the only metabolite with a significant group difference after FDR correction. Positive trends were observed between the sarcopenia *z* score and very long-chain fatty acids as well as dicarboxylic acid carnitines. Multivariate analysis showed citrulline, non-esterified fatty acid 26:2, and decanedioyl carnitine as the top three metabolites according to the variable importance in projection using oPLS-DA and loadings weight by sPLS-DA. Metabolite set enrichment analysis showed carnitine palmitoyltransferase deficiency (II) as the highest condition related to the metabolome.

**Conclusions:**

We observed a difference in the plasma metabolic profile in association with different measures of sarcopenia, which identifies very long-chain fatty acids, Carn.DC and citrulline as key variables associated with the disease severity. These findings point to a potential link between sarcopenia and mitochondrial dysfunction and portraits a number of possible biochemical pathways which might be involved in the disease pathogenesis.

**Supplementary Information:**

The online version contains supplementary material available at 10.1186/s13395-022-00312-w.

## Background

Sarcopenia is one of the most predominant musculoskeletal diseases of the elderly, defined as age-related progressive and generalized loss of muscle mass with a simultaneous reduction in muscle strength and/or function [1, 2]. Sarcopenia not only poses a risk for loss of activity of daily living, negatively impacting the quality of life, but also is associated with a high risk of falls, fractures, hospitalization, and mortality [3, 4]. Thus, it can be related to serious social and economic implications, reflected in the high health care costs [5, 6]. A better understanding of the factors underlying this condition may offer subsidies for treatment strategies. However, this can be complicated as sarcopenia is a multifactorial disease, which can be attributed to several factors and the elemental biological mechanisms are yet not clearly elucidated. Metabolomics is an omics science based on identifying and measuring the small molecule substrates, intermediates, and products of cell metabolism, globally known as “the metabolome.” It is a powerful approach, since the metabolome directly reflects the underlying biochemical activity and the physiological state, thus best representing the molecular phenotype. Therefore, metabolomics can be a valuable tool for understanding the metabolic disruptions associated with sarcopenia and can be also correlated with the severity of the disease.

In 2019, The European Working Group on Sarcopenia in Older People (EWGSOP2) published the revised consensus on the use of low muscle strength as the primary criterion for the identification of sarcopenia, with the diagnosis further confirmed by the presence of low muscle quantity or quality and physical performance as an indicator of severity [7–9]. Based on these criteria, sarcopenia was defined as the combination of reduced handgrip strength and loss of skeletal muscle mass, calculated as skeletal muscle index (SMI) [7, 9]. A z-transformation of both values represents the degree of sarcopenia on a metric scale.

The aim of this study was to examine the association between sarcopenia, defined as reduced handgrip strength and loss of skeletal muscle mass as the primary criterion of sarcopenia, as well as the SMI, with the plasma metabolic profile of sarcopenic patients, measured using a targeted metabolomics platform. Data were also provided on somatotropic axis parameters and relevant associations with the metabolic profile have been additionally investigated. The resulting associations were then used to draw a picture of the biochemical pathways involved in the disease pathogenesis with a potential link between sarcopenia and mitochondrial dysfunction.

## Methods

### Patient recruitment

This metabolomics study was performed as a secondary analysis on plasma samples obtained from 22 hip fracture patients of both sexes undergoing surgery. Briefly, patients aged over 70 years with a proximal hip fracture of the femur undergoing surgery were recruited from November 2017 to March 2019. Patients were excluded if they suffered from specific neuromuscular diseases (myasthenia gravis, muscular dystrophy, ALS, polio), severe dementia, chronic inflammatory disease (e.g., Crohn’s disease, ulcerative colitis, rheumatoid arthritis with systemic anti-inflammatory therapy), or have been subject to systemic corticosteroid therapy (above 7.5mg per day), or cancer therapy in the last 5 years. All participants provided written informed consent before enrolment. Informed consent was taken before surgery with enough time to think about participation. In case of concerns of the patient, the patient was not included.

### Patient data

The collected data includes information on the demographic, family, and socioeconomic characteristics, alcohol intake, smoking, and comorbidities as well as anthropometry (weight, height, BMI, fat mass (FM), and fat mass index (FMI). The anthropometric measurements were obtained by physical examination of the study participants by trained study personnel. Bio-Impedance Analysis (BIA; BIA 101, Akern, Florence, Italy) was performed after surgery and used for measuring lean mass. Measurements were taken under standard conditions, with the patient in a supine position and surface electrodes placed on the wrist and ankle contralateral to the side of the fracture. Appendicular lean mass (aLM) was estimated using the equation developed by Sergi et al. [10]. The skeletal muscle index [SMI, (kg/m^2^)] was calculated by dividing aLM by body height squared. Assigned cutoffs of 7 kg/m^2^ in men and 5.5 kg/m^2^ in women were used to define low SMI. Handgrip strength was assessed with a Saehan DHD-1 Digital Hand Dynamometer, with the patient lying supine. The maximal value of three consecutive measurements of both hands was used for the analysis. Similar to SMI, handgrip strength was defined as low, if below 27 kg and 16 kg, in men and women, respectively [7]. A *z* score combining handgrip strength and muscle mass was calculated separately for men [*z* score sarcopenia_men_ = [(27–handgrip strength)/SD (handgrip strength)] + [(7.0–SMI)/SD (SMI)] and women [*z* score sarcopenia_women_ = [(16–handgrip strength)/SD (handgrip strength)] + [(5.5–SMI)/SD (SMI)]. Z-transformation of both values represents the degree of sarcopenia on a metric scale. The higher the *z*-score, the more sarcopenic the patient. Data were provided on the insulin growth factor (IGF) axis parameters including IGF-1, insulin growth factor binding protein 3 (IGFBP3) and IGF1/IGFBP3 ratio.

### IGF-I and IGFBP3 measurement

Blood samples for measurement of serum concentrations of IGF-I and IGFBP3 were centrifuged and serum was stored at −80°C until analysis. Serum hormone concentrations (ng/ml) of IGF-I and IGFBP3 were measured at the Endocrine Laboratory of the University Hospital Munich using the IDS-iSYS automated chemiluminescent assay system (Immunodiagnostic System Ltd., Boldon, England, UK). Validation data for all assays and reference intervals have been published elsewhere [11, 12]. The assays are calibrated against the latest recombinant standards (02/254 for IGF-I).

### Metabolomic measurements

For the metabolomic measurements, plasma samples were obtained after centrifugation of blood samples collected from patients in EDTA tubes, then stored at −80°C until analysis. Overall, a targeted metabolomics approach was applied for measuring a total of 300 metabolites in the patients’ samples at the Dept. of Paediatrics, LMU Munich. Concentrations were calculated in μmol/l. The measured metabolites belonged to the following classes:

#### Amino acids

Twenty-two amino acids including alanine (Ala), arginine (Arg), asparagine (Asn), aspartic acid (Asp), glutamine (Gln), glutamic acid (Glu), glycine (Gly), histidine (His), isoleucine (Ile), leucine (Leu), lysine (Lys), methionine (Met), phenylalanine (Phe), serine (Ser), threonine (Thr), tryptophan (Trp), tyrosine (Tyr), valine (Val), citrulline (Cit), ornithine (Orn), and proline (Pro) were analyzed in plasma samples obtained from patients by ion-pair liquid chromatography with tandem mass spectrometry (HPLC-MS/MS ) as previously described by Harder et al. [13].

#### Acyl carnitines

Carnitines (free carnitine (Carn) and acylcarnitine (Carn.a)) were analyzed using a modified method from Giesbertz et al. [14]. Briefly, proteins of 50 μL plasma samples were precipitated by a tenfold amount of methanol including isotopic labeled internal standards D3-Carnitine C2 (DLM-754-PK, Cambridge Isotope Laboratories), D3Carnitine C8 (DLM-755-0.01, Cambridge Isotope Laboratories), and D3-Carnitine C16 (DLM-1263-0.01, Cambridge Isotope Laboratories). After centrifugation, 50 μL of the supernatant was evaporated to dryness under a gentle stream of nitrogen at 40 °C. The residuals were re-dissolved in 50 μL hydrogen chloride-1-butanol solution, and derivatization was conducted at 60 °C for 10 min shaking at 600 rpm. Thereafter, the hydrogen chloride-1-butanol solution was evaporated to dryness and the residuals re-dissolved in 50 μL methanol. The butylated acylcarnitines were separated on a 1200-SL HPLC system (Agilent Technologies, Waldbronn, Germany) equipped with a degasser, pump, autosampler, column oven, and a 150 × 2.1 mm Kinetex® reversed-phase column with 2.6 μm particles (Phenomenex, Torrance, USA). Mobile phase A consisted of 5mM ammonium acetate in water and mobile phase B consisted of 333 μL 7.5 M ammonia acetate in 1 L methanol/ acetonitrile/isopropanol (1:4:5). The mass spectrometric detection was performed on a hybrid triple quadrupole mass spectrometer (4000 QTRAP, AB Sciex, Darmstadt, Germany) with a Turbo Ion source operating in negative ESI mode.

#### Non-esterified fatty acids (NEFA)

Sixty-three non-esterified fatty acids were measured in patients’ samples, using HPLC-MS/MS run in negative ESI mode as described previously by Hellmuth et al. [15]. The same formula CX:Y was used to indicate the chain length as well as the number of double bonds.

#### Bile acids

A new method for bile acids analysis was developed and validated using HPLC-MS/MS. The method description is presented in the supplementary material (Supplementary M1). Briefly, seventeen bile acids were measured, including cholic acid (CA), chenodeoxycholic acid (CDCA), deoxycholic acid (DCA), lithocholic acid (LCA), glycocholic acid (GCA), taurocholic acid (TCA), glycochenodeoxycholic acid (GCDCA), taurochenodeoxycholic acid (TCDCA), glycodeoxycholic acid (GDCA), taurodeoxycholic acid (TDCA), glycolithocholic acid (GLCA), taurolithocholic acid (T LCA), taurocholic acid 3-sulfate (TCA-3S), and taurolithocholic acid 3-sulfate (TLCA-3S).

### Quality control

Due to the limited number of samples, only one batch was used in all analyses and quality control samples (QC) were used to check the within-batch variations (intra-batch CV% = 20%). Six QC prepared by pooling aliquots of all available study samples were consistently measured at regular intervals within the batch at the beginning, middle, and end of the batch. Measurements greater than 1.5 standard deviations (SD) away from the next closest measurement were considered as outliers and subsequently set to NA (not available). Measurements with >50% missing values were excluded.

### Statistical analysis

#### Descriptive statistics

The demographic and phenotypic characteristics of the study participants, including age, sex, BMI, comorbidities (diabetes mellitus, rheumatoid arthritis, thyroid, and parathyroid dysfunction, spine diseases, chronic lung diseases, kidney diseases, cancer, diarrhea intolerance), smoking, alcohol and drug intake (proton pump inhibitors (PPI), corticosteroids, anti-estrogenic therapy, tranquilizers), mobility problems, dizziness, stumbling, falls during the preceding year to the study, and activities as sports, daily outdoor activities, were summarized as mean (SD) and proportions for continuous and categorical variables, respectively. Wilcoxon rank-sum test and Fisher’s exact test were used to investigate the differences between groups for numerical and categorical variables, respectively. Results are shown in Table 1.

**Table 1:** Study population characteristics

Characteristics	Non-sarcopenic ***(n=14)***	Sarcopenic ***(n=8)***	***P*** value
Age	78.4±8.1	81.4+6.3	ns
Sex			
Male	11 (79%)	6 (25%)	ns
Female	3 (21%)	2 (75%)	
**Body measures**			
Body weight (kg)	76.1±15.8	64.6±15.2	ns
Body height (cm)	167.6+8.5	170.5±10.1	ns
Body mass index (BMI)	26.9±4.2	22.2±4.5	0.02
Fat mass (kg)	27.0±10.7	17.3+6.5	ns
Fat mass percent (FM%)	33.5±6.0	26.2±7.4	ns
Free fat mass (kg)	51.5±10.3	47.3±10.7	ns
Free fat mass percent (FFM%)	66.5±6.0	73.8±7.4	ns
**Measures of sarcopenia**			
Grip strength	27.3±8.4	18.1±4.9	0.01
Appendicular lean mass (aLM)	18.8±4.3	18.4±4.8	ns
Skeletal muscle index (SMI)	6.6±0.9	6.3±1.3	ns
Sarcopenia *z* score	−1.67±1.21	0.89±1.06	<0.001
**Co-morbidities**			
Smoking	2 (14%)	1 (13%)	ns
Chronic lung disease	0 (0%)	1(13%)	ns
Kidney disease	2 (14%)	2 (25.0%)	ns
Rheumatoid arthritis (without systemic anti-inflammatory therapy)	3 (21%)	1 (12.5%)	ns
Cancer (> 5 years in their anamnesis)	3 (21%)	4 (50%)	ns
Parathyroid gland dysfunction	0 (0%)	1 (13%)	ns
Thyroid gland dysfunction	2 (14%)	1 (13%)	ns
Diabetes mellitus (DM)	2 (14%)	2 (25%)	ns
Spine disease	1 (7%)	0 (0%)	ns
Diarrhea	0 (0%)	3 (38%)	<0.05
**Medication**			
Regular drug intake	13 (93%)	7 (88%)	ns
Corticosteroids (<7.5mg per day)	4 (29%)	2 (25%)	ns
DM drug treatment	1 (7%)	0 (0%)	ns
Proton pump inhibitors (PPI)	7 (50%)	4 (50%)	ns
Tranquilizers	3 (21%)	2 (25%)	ns
**Mobility/balance problems**			
Mobility problems	0 (0%)	3 (38%)	<0.05
Stumbling	3 (21%)	2 (25%)	ns
Dizziness	5 (36%)	3 (38%)	ns
Walking aid	2 (14%)	6 (75%)	<0.01
Household independent	3 (21%)	4 (50%)	ns
Needs help in shopping	1 (7%)	5 (63%)	<0.05
Number of falls in the preceding year	2 (14%)	6 (75%)	<0.01
**Activities**			
Daily outside activity	1 (7%)	3 (38%)	ns
Regular sports	9 (64%)	2 (25%)	ns
**Surgeries**	0 (0%)	2 (12.5%)	ns
**IGF axis parameters**			
IGF1	68.4±19.0*	53.8+28.5	ns
IGFBP3	1588±430*	1419±567	ns
IGF1/IGFBP3	16.9±5.0	14.2+4.1	ns

*Values are expressed in “mean± SD range” or “absolute number (percentage)”

*ns* not significant at *P*<0.05

#### Data analysis

For the metabolomics data, after normalization and scaling, linear regression models were used to study the associations between plasma metabolite levels and different measures of sarcopenia (sarcopenia *z* scores and SMI) using the sarcopenia measures as the outcome and the plasma metabolites as the independent variables. Models were initially adjusted using potential confounders including age, sex, BMI, smoking, alcohol intake, and comorbidities such as cancer; however, it was noticed that the associations between the metabolite levels and the sarcopenia measures were not appreciably influenced by the inclusion of these confounders, hence they were not included in the models, especially considering the small sample size. Volcano plots were used to depict the results of the models with *β* on *x*-axis and |log10 (*P*)| values on *y*-axis indicating the sign, magnitude, and strength of the association, respectively. False discovery rate (FDR) [16] was used to minimize the occurrence of false positives, a common issue in multiple testing. Nevertheless, we also inspected associations with uncorrected *P* values for further interpretation, because of the exploratory nature of the analysis and regarded some as potentially meaningful differences, principally if they are common among the same metabolite class or subclass and share the same tendencies. These associations were referred to as trends albeit not significant after FDR correction due to the low statistical power. The cutoff for uncorrected *P* values was depicted as a red dotted line in the volcano plot. Additionally, sarcopenic and non-sarcopenic patient groups were defined using the sarcopenia *z* score cutoff values. To explore the group differences, unadjusted comparisons using multiple univariate tests were performed within the Metaboanalyst 5.0 software which includes fold change (FC) analysis and Wilcoxon rank-sum test. Then, a combination of both tests was used to produce a volcano plot using a cut-off of 1.5 and 0.05 for FC and *P* value, respectively [17]. Concomitantly, for class discrimination and identification of metabolites responsible for group separation, multivariate analyses were also conducted including principal component analysis (PCA), partial least squares–discriminant analysis (PLS-DA), and orthogonal partial least squares–discriminant analysis (oPLS-DA).

Metabolite set enrichment analysis (MSEA) was also performed using the metabolomics data sets, and the pathway was considered significantly enriched if *P* values were smaller than 0.05 and those significant after FDR correction were inspected. Both multivariate analysis and pathway enrichment analysis were carried out also using Metaboanalyst 5.0. Causal effect relationships involved in sarcopenia were investigated using Mediation Analysis by PLSSEM [18–20] using SmartPLS software. First, factor analysis was performed for the selection of indicator variables most associated with the relevant latent variables for each of the metabolite class (AA, NEFA, BA, Carn.a, TCA). Then bias-corrected and accelerated bootstrap was conducted to test the statistical significance of the investigated pathways using 0.05 as a significance level and the total effects, total indirect effects, and specific indirect effects were calculated.

## Results

The demographic and phenotypic characteristics of the study participants are described in detail in Table 1. Overall, the 22 patients included 17 men and 5 women aged 79.5±7.5 years. Eight patients were found to suffer sarcopenia as per the EWGSOP2 guidelines, while the remaining 14 subjects were non-sarcopenic. Regarding the definition of sarcopenia, which is the combination of low handgrip strength and low muscle mass, handgrip strength contributes to a higher degree than muscle mass in the patients investigated. This is also reflected in a greater sarcopenia *z* score. The sarcopenic group was aged 81.4±6.3 years while the non-sarcopenic group was aged 78.4±8.1 y. We noticed lower levels of insulin growth factor (IGF-I) and IGF-I/insulin growth factor binding protein 3 (IGFBP3) in the sarcopenic group relative to the non-sarcopenic group.

### Association between measures of sarcopenia and the plasma metabolite levels

**Fig. 1:**
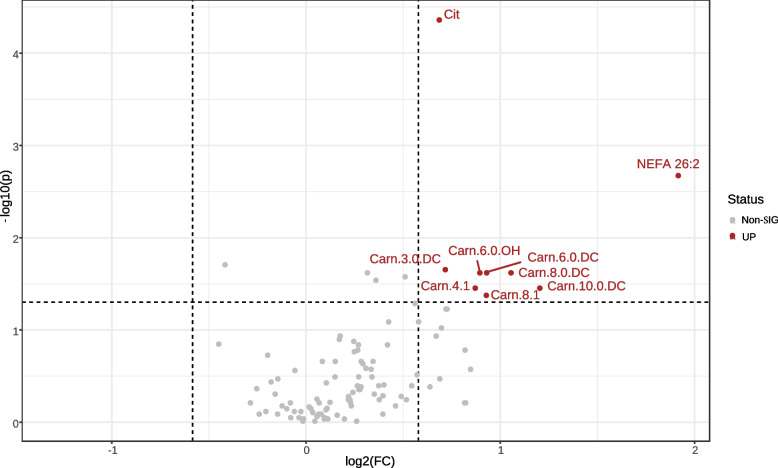
Volcano plot depicting significant metabolites between the sarcopenic and non-sarcopenic groups using a fold change (FC) threshold of 1.5 and *t* test threshold of 0.05. The log-transformed FC and *P* values are represented on *x*- and *y*-axes, respectively. The red circles represent features above the selected thresholders

Linear regression models were used to investigate the associations between the sarcopenia *z* score SMI, and maximum handgrip strength as measures of sarcopenia and the plasma metabolite levels. For sarcopenia *z* score, the amino acid citrulline (Cit) was the only metabolite found significant after false discovery rate (FDR) correction, which stood out as highly significant (*p*<0.001). Positive trends (not significant after FDR correction) were observed between the sarcopenia *z* scores and long as well as very long-chain non-esterified fatty acid (VLC-NEFA), namely NEFA 16:3, NEFA 24:2, NEFA 26:1, and NEFA 26:2) as well as dicarboxylic acid carnitines (Carn-DC) (Supplementary Table 1). Volcano plot for the group differences between the sarcopenic and non-sarcopenic groups using FC analysis and Wilcoxon rank-sum test identified Cit, 4 Carn-DC, namely Carn.3.0.DC, Carn.6.0.DC, Carn.8.0.DC, Carn.10.0.DC in addition to Carn.4.1, Carn.8.1, and Carn.6.OH as well as NEFA 26:2, as shown in the volcano plot (Fig. 1) of which only Cit remained significant after FDR correction (*P*<0.01). For the SMI, we found a negative association between SMI and mid to long-chain acylcarnitines (Carn.a) as well as mid-chain NEFA. Additionally, negative trends (not significant after FDR correction) were found with primary bile acids (BA): cholic acid (CA), and chenodeoxycholic acid (CDCA) and a tertiary BA: ursodeoxycholic acid (UDCA) while positive trends with secondary BA taurolithocholic acid (TLCA) and glycolithocholic acid GLCA and one primary BA: taurocholic acid (TCA) and the two amino acids (AA): His and Val (Supplementary Table 1). None of these associations were significant after FDR correction (Supplementary Table 1). Similar to the SMI, negative association between the maximum handgrip strength and mid to long-chain Carn.a (Carn.12.0, Carn.12.1, Carn.14.1, Carn.14.2, and Carn.16.1). However, the most striking observation was the positive trends observed between the handgrip strength and several long and very long-chain NEFA (VLC-NEFA), represented in 22 NEFA species as shown in Supplementary Table 1 albeit not significant after FDR correction. Similar to the SMI, positive associations were found between the TCA, TLCA, and the maximum handgrip strength. Additionally, TCA-3S and TCDCA were also positively associated.

#### Multivariate analysis

**Fig. 2:**
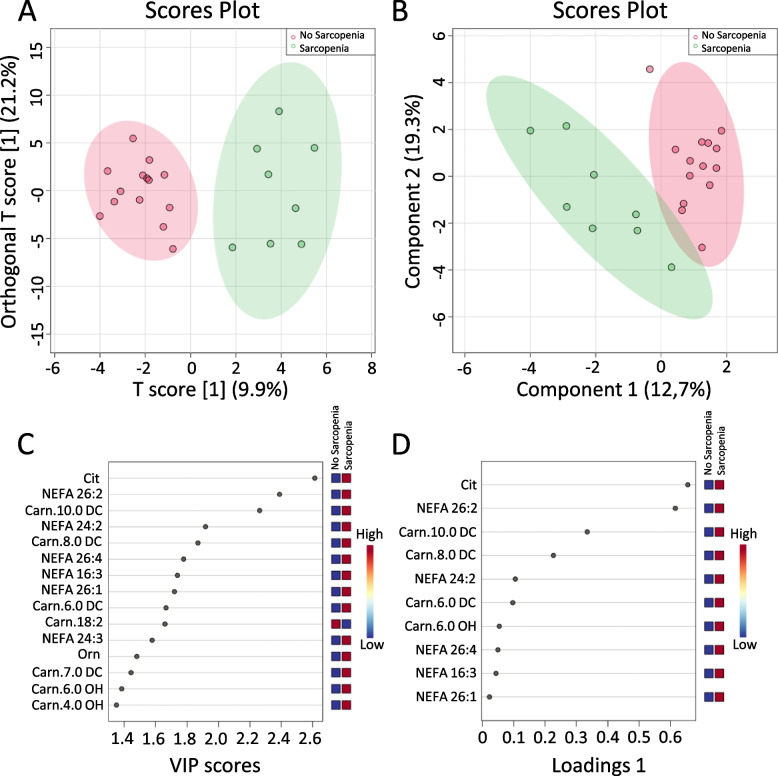
**A** Score plot from the orthogonal partial least square discriminant analysis (oPLS-DA) showing complete group separation from non-sarcopenic (red circles) and sarcopenic (green circles) subjects (95% confidence ellipses are shaded around each group). **B** Score plot from the sparse partial least square discriminant analysis (sPLS-DA) depicting group separation to a great extent on PC 1 (*x*-axis) and to a large extent using PC1 and PC2 from non-sarcopenic (red circles) and sarcopenic (green circles) subjects (95% confidence ellipses are shaded around each group). **C** Variable importance in projection (VIP) scores plot from the orthogonal partial least square discriminant analysis (oPLS-DA) presenting the metabolites driving the separation between the non-sarcopenic group and the sarcopenic group. **D** Loadings plot from the sparse partial least square discriminant analysis (sPLS-DA) ranking the metabolites with the highest loadings weight in the model responsible for driving the separation between the non-sarcopenic group (0) and the sarcopenic group [1]

Multivariate models were applied to evaluate the separation between sarcopenic and non-sarcopenic patient groups. Among the different approaches described in the methods section, orthogonal partial least square discriminant analysis (oPLS-DA) and sparse partial least square discriminant analysis (sPLS-DA) were found to be the most efficient models. Complete group separation was obtained using oPLS-DA (Fig. 2A). Cit, NEFA 26:2, and Carn 10.DC were recognized as the top metabolites according to the variable importance in projection (VIP), driving the separation using this model (Fig. 2C). sPLS-DA was found to separate the two groups to a great extent on the first principal compound (PC1) (Fig. 2B). Similar to oPLS-DA, Cit, NEFA 26:2, and Carn 10.DC were recognized as metabolites with the highest loading weights in the model, in addition to other relevant metabolites, such as other Carn.DC (Carn 8.DC and Carn 6.DC) and very long-chain fatty acids (VLC-FA) as NEFA 24:2 (Fig. 2D).

#### Metabolite set enrichment analysis

**Fig. 3:**
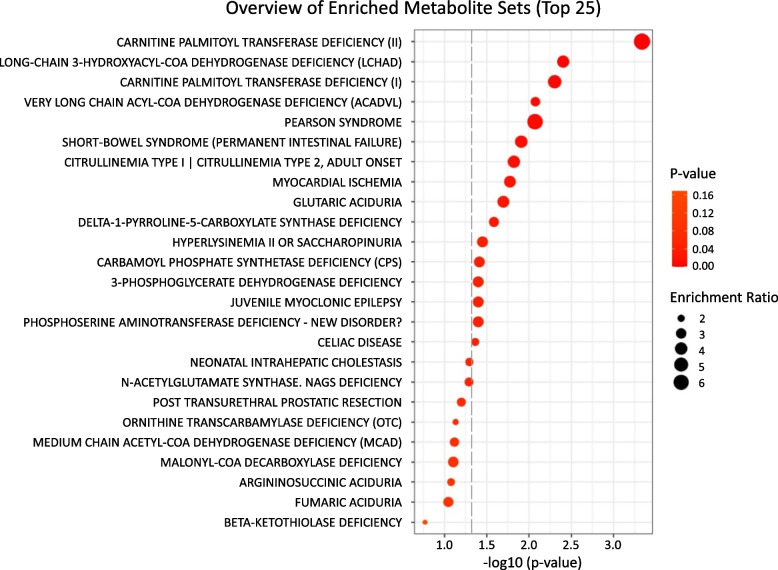
Quantitative enrichment analysis (QEA) overview presenting the top 25 related metabolic pathways ranked according to the *P* value. Enrichment ratio is computed by hits/expected, where hits = observed hits; expected = expected hits. Dashed line indicates *P*=0.05 (-log10 (*P* value)= 1.3

Quantitative enrichment analysis (QEA) (Fig. 3) showed that the top enriched metabolite sets identified by the difference in the metabolic profiles between the sarcopenic and the non-sarcopenic groups were carnitine palmitoyltransferase deficiency (II), long-chain -3-hydroxy acyl-coA dehydrogenase deficiency (LCHAD), carnitine palmitoyltransferase deficiency (I), very long-chain acyl co-A dehydrogenase deficiency (VLCAD), and Pearson syndrome (uncorrected *P*<0.05). All the conditions related to these metabolite sets share the common feature of being mitochondrial respiratory chain disorders (Supplementary Table 2).

### Associations between the somatotropic axis parameters and the metabolome

Negative trends were found between IGF-I and three VLC-FA species (NEFA 26:2, NEFA24:4, NEFA 24:2), and Cit. Fewer associations were found between the metabolome and IGFBP3, where it was only negatively associated with two metabolites: Carn.a 20:0 and CA. As for the ratio IGF-I/IGFBP3, it was the most associated with the metabolome, especially the NEFA. This was evident in the multiple negative trends observed between the NEFA, specifically the LC- and the VLC-NEFA and the ratio IGF-I/IGFBP3 including NEFA 13:0, NEFA 17:1, NEFA 16:1, NEFA 14:1, NEFA 12:1, NEFA 19:1, NEFA 16:3, NEFA 16:0, NEFA 22:2, NEFA 18:2, NEFA 14:2, NEFA 24:4, NEFA 22:4, NEFA 14:0, NEFA 24:3, NEFA 22:3, and NEFA 24:2. This in addition to Carn.a 3:0 and a BCKA, 3-methyl-2-oxo butanoic acid which were also negatively associated. None of the abovementioned associations between the somatotropic axis parameters and the metabolome were found significant after correction for FDR (Table 2).

**Table 2:** Results from the linear models for the associations between the plasma metabolites with (A) insulin-like growth factor I (IGF-I) and (B) IGF-I/insulin-like growth factor-binding protein (IGFBP3) in the study population

Class	Metabolite	Regression coefficient (***β***)	***P*** value	***P*** value (after FDR correction)
**A. Insulin-like growth factor I (IGF-I)**				
** AA**	Cit	−0.024	0.016	ns
** NEFA**	NEFA.26:2	−0.021	0.043	ns
	NEFA.24:4	−0.02	0.045	ns
	NEFA.24:2	−0.02	0.05	ns
**B. IGF-I/insulin-like growth factor BP3**				
** NEFA**	NEFA.13:0	−0.181	0.001	ns
	NEFA.17:1	−0.165	0.004	ns
	NEFA.16:1	−0.164	0.004	ns
	NEFA.14:1	−0.163	0.005	ns
	NEFA.12:1	−0.16	0.006	ns
	NEFA.19:1	−0.156	0.007	ns
	NEFA.16:3	−0.15	0.011	ns
	NEFA.16:0	−0.149	0.011	ns
	NEFA.22:2	−0.143	0.015	ns
	NEFA.18:2	−0.143	0.016	ns
	NEFA.14:2	−0.141	0.017	ns
	NEFA.24:4	−0.138	0.02	ns
	NEFA.22:4	−0.136	0.022	ns
	NEFA.14:0	−0.133	0.026	ns
	NEFA.24:3	−0.127	0.034	ns
	NEFA.22:3	−0.121	0.047	ns
	NEFA.24:2	−0.127	0.034	ns
** Carn.a**	Carn.3.0	0.125	0.038	ns

*AA* amino acids, *Carn.a* acylcarnitines, *NEFA* non-esterified fatty acids. *P* values as *ns* non-significant

#### Mediation analysis by PLSSEM

Given that specific metabolite species were commonly associated with both, IGF-I and sarcopenia, we hypothesized that a somatotropic axis may impact sarcopenia mediated by metabolic changes. To test this hypothesis, PLSSEM was used using each of the metabolite class sets as mediators (NEFA, BA, AA, and Carn.a). We noticed that none of the direct or the indirect effects from IGF-I to sarcopenia was significant using any of the metabolite classes except VLC-FA. The specific indirect effects from IGF-I to sarcopenia mediated by VLC-FA were found significant (*P*<0.05) indicating the role of the somatotropic axis shares with VLC-FA in sarcopenia. A diagram depicting the path model from IGF-I to sarcopenia mediated by NEFA and the results of pathways significance (path coefficients, specific indirect effects, and total indirect effects) are depicted in Supplementary Figure 1 and Supplementary Table 3, respectively.

## Discussion

A peculiar profile to this study was the higher levels of circulating LC- and VLC-FA, and their downstream metabolites (Carn D.C., especially the mid-chain ones) along with the remarkably higher levels of the Cit in patients with sarcopenia relative to non-sarcopenic ones (Supplementary Table 1). This was evident in the trends for higher levels of these metabolites in association with sarcopenia *z* score either using the linear regression models (where the sarcopenia *z* score is treated as a continuous variable) or using univariate/multivariate methods employing OPLS-DA/SPLS-DA, where the patients were divided into sarcopenic and non-sarcopenic groups. The most characteristic compounds detected as top significant metabolites were Cit, NEFA 26:2, Carn 10. DC in addition to other VLC-NEFA and Carn.DC as previously discussed in the results section (Figs. 1 and 2).

The mechanism behind the accumulation of Carn.DC is probably relevant to dicarboxylic acids produced by the ω-oxidation of long-chain fatty acid (LC-FA) and VLC-FA [21]. The corresponding activated CoA esters undergo some cycles of β-oxidation in the peroxisome up to a certain point, probably C10-dicarboxylyl-CoA, which is transported to the mitochondria as Carn. 10.DC [22]. Therefore, it would be fair to hypothesize that the elevation in levels of Carn.DC is a consequence of the elevated levels of VLC-NEFA. Complementary to this picture, we observed a trend for higher levels of mid to LC- NEFA and LC- Carn.a in association with SMI despite not being significant after FDR correction. This metabolic picture is common in mitochondrial long-chain LC-FA oxidation disorders, as carnitine-acylcarnitine translocase deficiency, which involves a transport defect of LC-FA across the mitochondrial membrane, causing an elevation in the levels of plasma LC-FA and monocarboxylic LC-Carn.a [23] [22]. The excess of LC-FA and VLC-FA (C20 or more) synthesized by the elongation of precursor LC-FA via reactions catalyzed by microsomal elongation enzymes is the first subject to ω-oxidation and producing dicarboxylyl-CoA esters which then undergo β-oxidation (chain shortening) in peroxisomes but only to a limited extent, producing C10-dicarboxylyl-CoA, which cannot enter the mitochondrion, leading to its accumulation, in accordance with our results [22]. Our hypothesis is supported by the results of the pathway enrichment analysis, showing that the top enriched pathways identified between the sarcopenic and the non-sarcopenic groups are related to mitochondrial disorders. Even though the exact mechanisms underlying sarcopenia are not fully understood, there is evidence that the accumulation of damaged mitochondria could trigger motor neuron and muscle fiber death [24]. Furthermore, studies have identified mitochondrial as one of the central players contributing to the pathogenicity of the disease [25, 26].

Previous studies showed that mitochondrial dysfunction arising from the abnormal accumulation of mitochondrial DNA induced the early appearance of several age-related phenotypes, including sarcopenia, in mice [27–29]. Several reasons can contribute to mitochondrial dysfunction/damage including ROS-induced damage. ROS production increases with aging due to the decreased levels of antioxidant enzymes and might be secondary to age muscle denervation [24].

Another observation that stood out was the significantly higher levels of Cit in sarcopenic relative to non-sarcopenic subjects, which was the only metabolite found significant after FDR correction. Cit was previously linked to muscle wasting through NO-induced stress being a precursor in nitric oxide (NO) metabolism [30]. iNOS converts l-arginine to Cit, releasing NO, which reacts with the reactive oxygen species (ROS) (superoxide anions O_2_-) forming the toxic molecule peroxynitrite (ONOO-), leading to oxidative stress and muscle fiber loss, a mechanism triggered by tumor necrosis factor-alpha (TNFα) [31, 32]. The implication of the iNOS/NO pathway in TNFα-induced muscle atrophy was previously reported in the literature despite that the detailed mechanism remains unclear [31, 33, 34]. Some studies demonstrated that NO and peroxynitrite levels were found to decrease levels of transcription factors involved in myogenesis and skeletal muscle health [31, 35].

Our findings are strengthened by the consistent results obtained when using either univariate or multivariate analysis, which both showed VLC-FA and Carn.DC as well as Cit as key variables associated with sarcopenia as well as the complementary findings from SMI and handgrip strength which further supported the findings. Although SMI and maximum handgrip strength did not show associations with the exact same set of metabolites, the findings complement those obtained using sarcopenia *z* score whether by univariate or multivariate analysis, such as the negative association between SMI and mid to long-chain acylcarnitines (Carn.a) and mid- and LC- NEFA. This supports the assumption of an impaired mitochondrial function affecting the β-oxidation of the fatty acids and leading to the accumulation of LC- Carn.a and mid-chain NEFA, thus suggesting incomplete oxidation (Supplementary Table 1). Another class that popped up with SMI and maximum handgrip strength are bile acids, with two common findings which are the positive associations between TCA and TLCA and both measures of sarcopenia (Supplementary Table 1). Other bile acids were uniquely associated either to SMI or maximum handgrip strength. For SMI, primary bile acids (CA, CDCA) and tertiary (UDCA) showed negative trends, in contrast with conjugated bile acids (TLCA, GLCA) while positive trends were found between TCA-3S and TCDCA, and maximum handgrip strength. For the sarcopenia z-score, the only bile acid associated was CA which was positively associated with. A recent study demonstrated the atrophic effects of the two bile acids (DCA and CA) on skeletal muscle fibers through TGR5, a plasma membrane G-protein-coupled receptor, in association with increased levels of oxidative stress and protein catabolic pathways [36]. These findings are in line with previous studies on the role of DCA in protein catabolism and energy consumption through TGR5 activation, thus suggesting it as a potential biomarker of sarcopenia, arising in patients with advanced non-alcoholic fatty liver disease (NAFLD) [37]. Another study reported an association between serum bile acids and skeletal muscle volume (SMV) in NAFLD patients [38]. In this study, DCA levels were negatively correlated with SMV of the upper and lower limbs and total SMV while CDCA levels were positively correlated with an increased SMV of the lower limbs. Hepatocytes exposed to high levels of bile acids were shown to exhibit changes in mitochondrial function, including reduced electron transport, impaired mitochondrial respiration, mitochondrial swelling, and outer membrane permeabilization, known as mitochondrial permeability transition (MPT). All these events can eventually cause cell death [39–41]. Oxidative stress has been recognized as a primary factor in bile acid-induced MPT which can be responsive to antioxidant treatment [42]. We speculate that the opposite directions of associations between unconjugated and conjugated bile acids with SMI may reflect reduced conjugation of BA in the peroxisomes, which might be secondary to the change in the mitochondrial function affecting the peroxisomes. Evidence has been provided, that peroxisomes and mitochondria exhibit a close functional interplay and coordinated biogenesis to address certain conditions and demands [43].

Regarding the associations between IGF-I and the metabolome, we observed the involvement of the same metabolite classes, VLC-FA (NEFA 26:2, NEFA24:4, NEFA 24:2) and Cit (Table 2). As for the ratio IGF-I/IGFBP3, it was the most associated with several LC- and the VLC-NEFA (Table 2) which might suggest the implication of IGF-I in the mechanisms related to sarcopenia. We tried to test this hypothesis through PLSSEM. PLSSEM is regarded as an effective tool for conducting exploratory research to develop or extend theory and is particularly useful with small sample sizes which makes a good choice in the current analysis [20]. Despite the small number of samples, we observed that specific indirect effects from IGF-I to sarcopenia mediated by VLC-FA were found significant (*P*<0.05), which further supports our hypothesis.

Lower levels of IGF-I have been linked to mitochondrial dysfunction in aging rats characterized by permeabilization, loss of membrane potential, increased proton leak rates, intramitochondrial free radical production, and a reduction of ATPase and complex IV activities which were ameliorated by exogenous administration of IGF-I in those aging rats [44, 45]. IGF-I therapy was found to improve the oxidative stress damage observed in aging mice with mitochondrial dysfunction [46] and normalize the antioxidant enzyme activities [44, 45]. Thus, these findings suggest that IGF-I has a cytoprotective effect closely related to mitochondrial protection, decreasing free radical production, oxidative damage, and apoptosis, and an increase of ATP production [45]. Impaired insulin action was linked to dysregulation of mitochondrial function, considering that insulin signaling is a prerequisite for mitochondrial DNA and protein synthesis and thus stimulating the mitochondrial oxidative capacity and ATP production [47, 48]. In line with this, deletion of insulin receptor (IR) and IGF-I receptor (IGF-IR) in the heart, was associated with the downregulation of genes of the mitochondrial electron transport chain and thus mitochondrial fatty acid β-oxidation in the heart [49, 50]. On the other hand, other studies demonstrated that IGF-I has no direct effect on lipid oxidation as a growth hormone which can directly stimulate fatty acid oxidation in an action not mediated by insulin-like growth factor-I [51]. We also tested this hypothesis but none of the direct or the indirect effects of GH on the lipid oxidation was found significant (results not shown). Therefore, our hypothesis that reduced levels of IGF-I might play a role in mitochondrial dysfunction is reflected in the elevation in VLC-FA and Carn.DC profiles which may play a role in sarcopenia. Our hypothesis is supported by previous reports on the decreases in growth hormone and plasma IGF-I with aging both in humans and animal models [52–54]. However, further studies are required to confirm these findings using large cohorts with a bigger sample size.

### Strengths and limitations

Based on our findings, we have drawn a picture on the biochemical pathways implicated in sarcopenia which may contribute to the disease pathogenesis and could potentially be regarded as biomarkers correlated with the severity of the disease. However, this study has some limitations which worth mentioning such as the small sample size, lack of information on the diet, and education, in addition to bias in subject selection. The study also applies a targeted metabolomics approach which may have missed some relevant metabolites. Accordingly, it can be considered as a proof-of-principle, model building pilot study for future larger studies targeting metabolic changes related to sarcopenia.

## Conclusions

In conclusion, we observed a difference in the plasma metabolic profile in association with different measures of sarcopenia, which identifies VLC-FA and Carn.DC as well as Cit as key variables associated with the disease severity. These findings point to a potential link between sarcopenia and mitochondrial dysfunction and portraits a number of possible biochemical pathways which might be involved in the disease pathogenesis. Large-scale studies can be used in the future to confirm the findings.

## Supplementary Information

**Additional file 1:** **Supplementary Table 1.** Chromatography and mass spectrometry parameters for the HPLC-MS/MS analysis of bile acids.

**Additional file 2: Supplementary Table 1.** Association of the metabolome with sarcopenia z-score SMI, and maximum grip strength.

**Additional file 3: Supplementary Table 2.** Result from Quantitative Enrichment Analysis.

**Additional file 4: Supplementary Table 3.** Results of pathway significance using mediation analysis by partial least squares-structural equation modeling (PLSSEM).

**Additional file 5: Supplementary Figure 1.** Path model by PLSSEM showing causal effect relationship from IGF-I to sarcopenia mediated by NEFA.

## Data Availability

The data underlying this article will be shared at reasonable request to the corresponding author.

## Abbreviations

AA: Amino acids
Ala: Alanine
Arg: Arginine
Asn: Asparagine
Asp: Aspartic acid
BMI: Body mass index
CA: Cholic acid
Carn: Free carnitine
Carn.a: Acylcarnitine
Carn-DC: Dicarboxylic acid carnitines
CDCA: Chenodeoxycholic acid
Cit: Citrulline
DCA: Deoxycholic acid
EWGSOP2: The European Working Group on Sarcopenia in Older People
FC: Fold change
FDR: False discovery rate
GCA: Glycocholic acid
GCDCA: Glycochenodeoxycholic acid
GDCA: Glycocholic acid
GLCA: Glycolithocholic acid
Gln: Glutamine
Glu: Glutamic acid
Gly: Glycine
His: Histidine
IGF: Insulin growth factor
IGFBP3: Insulin growth factor-binding protein 3
IGF-IR: Insulin growth factor—insulin receptor
Ile: Isoleucine
IR: Insulin receptor
LCA: Lithocholic acid
LC-FA: Long-chain fatty acid
Leu: Leucine
Lys: Lysine
Met: Methionine
MSEA: Metabolite set enrichment analysis
NEFA: Non-esterified fatty acid
NO: Nitric oxide
ONOO-: Peroxynitrite
oPLS-DA: Orthogonal partial least squares–discriminant analysis
Orn: Ornithine
PC: Principal compound
PCA: Principal component analysis
Phe: Phenylalanine
PLS-DA: Partial least squares discriminant analysis
Pro: Proline
QC: Quality control
QEA: Quantitative enrichment analysis
ROS: Reactive oxygen species
Ser: Serine
SMI: Skeleton muscle index
sPLSDA: Sparse least square discriminant analysis
TCA: Taurocholic acid
TCA-3S: Taurocholic acid 3-sulfate
TCDCA: Taurochenodeoxycolic acid
TDCA: Taurodeoxycholic acid
Thr: Threonine
TLCA: Taurolithocholic acid
TLCA-3S: Taurolithocholic acid 3-sulfate
TNFα: Tumor necrosis factor-alpha
Trp: Tryptophan
Tyr: Tyrosine
Val: Valine
VIP: Variable importance in projection
VLC-FA: Very long-chain fatty acids
VLC-NEFA: Very long-chain NEFA
