# Supplementary material for: Sarcopenia: investigation of metabolic changes and its associated mechanisms
Source: Skelet Muscle. 2023 Jan 19;13:2. doi: 10.1186/s13395-022-00312-w (PMC9850598; doi:10.1186/s13395-022-00312-w)
Supplement: Supplementary file 1 — Additional file 1: Supplementary Table 1. Chromatography and mass spectrometry parameters for the HPLC-MS/MS analysis of bile acids. [file 13395_2022_312_MOESM1_ESM.docx]

**Supplementary M1. Chromatography and mass spectrometry parameters for the HPLC-MS/MS analysis of bile acids**

**Bile acids method**

Seventeen bile acids were measured, including: cholic acid (CA) and chenodeoxycholic acid (CDCA), deoxycholic acid (DCA), lithocholic acid (LCA), glycocholic acid (GCA), taurocholic acid (TCA), glycochenodeoxycholic acid (GCDCA), taurochenodeoxycholic acid (TCDCA), glycodeoxycholic acid (GDCA), taurodeoxycholic acid (TDCA), glycolithocholic acid (GLCA), taurolithocholic acid (T LCA), tauro- cholic acid 3-sulfate (TCA-3S), and lithocholic acid 3-sulfate (TLCA-3S). Deuterated standards were used as internal standard for quantification, including CA-d4, DCA-d4, CDCA-d4, LCA-d4, UDCA-d4, GCA-d4, GCDCA-d4, TCA-d4, and TCDCA-d4.

**Plasma sample preparation**

50 µL of EDTA plasma samples were placed into a deep well plate followed by the addition of 450 µL of methanol containing 50 ng/mL of each internal standard. The samples were then mixed in a shaker at room temperature for 20 minutes at 900 rpm, cooled for 20 minutes at -20 °C, and filtered using a filter plate Chromafil® Multi 96 into a deep well plate by centrifugation at 1200 rpm for 10 min at 22°C. An aliquot of 50 µL was transferred into an AXIGEN 96 well full skirt PCR microplate, dried under nitrogen, and reconstituted in the initial mobile phase (36% of A and 65% of B) for HPLC-MS/MS analysis.

**Chromatography**

The analysis was conducted on a 1290 Infinity II high-performance liquid chromatography (HPLC) system (Agilent, Santa Clara, CA). Separation was achieved by reverse phase chromatography by injecting 5 µL using a Kinetex EVO C18 column (2,1 x 150 mm, 2,6 µm - Phenomenex) at 40 °C. The eluent comprises mobile phase A: H2O (10 mM ammonium acetate and 0.015% formic acid) and B: 60% acetonitrile, 30 % Methanol, and 10% isopropanol (10 mM ammonium acetate and 0.015% formic acid). Mobile phase gradient and flow rate are shown in **Table A**.

**Table A**. Chromatography gradient

| Time | Flow rate (µL / min) | A (%) | B (%) |
| --- | --- | --- | --- |
| 0.00 | 450 | 65 | 35 |
| 1.00 | 450 | 65 | 35 |
| 1.50 | 450 | 50 | 50 |
| 7.50 | 450 | 40 | 60 |
| 8.00 | 450 | 10 | 90 |
| 9.00 | 450 | 0 | 100 |
| 11.00 | 450 | 0 | 100 |
| 11.50 | 450 | 65 | 35 |
| 15.00 | 450 | 65 | 35 |

**Mass spectrometry**

MS measurements were performed on a QTRAP® 6500+ equipped with Turbo VTM source (SCIEX, Toronto, Canada) fully controlled by Analyst software (Version 1.7.0). The instrument was operated with electrospray ionization (ESI) in negative ion mode. Acquisition was performed in multiple reaction monitoring mode (MRM) with scheduled MRM using detection window of 120 sec, minimum dwell time of 3 ms and maximum dwell time: 250 ms. The source parameters ere: CUR= 50.00; TEM = 650.00 °C; GS1 = 40.00; GS2 = 70.00; CAD = Medium; IS = -4500.00 and EP = -10.00. The optimized transitions, parameters and retention time are presented in **Table B**.

**Table B**. Analytes ID, Q1 and Q3 transitions, retention time (RT), declustering potential (DP), collision energy (CE) and collision cell exit potential (CXP).

| ID | Q1 | Q3 | RT | DP | CE | CXP |
| --- | --- | --- | --- | --- | --- | --- |
| TCA-3S | 594.2 | 514.4 | 2.10 | -92 | -40 | -5 |
| TCA-3S_q2 | 594.2 | 594.2 | 2.10 | -90 | -11 | -5 |
| GUDCA | 448 | 74.0 | 3.33 | -115 | -76 | -9 |
| GUDCA_q2 | 448 | 386.0 | 3.33 | -110 | -48 | -9 |
| TUDCA | 498.8 | 80.0 | 3.34 | -122 | -105 | -10 |
| TUDCA_q2 | 498.8 | 123.8 | 3.34 | -200 | -74 | -5 |
| GCA | 464.4 | 74.0 | 3.55 | -121 | -77 | -5 |
| GCA_q2 | 464.4 | 402.0 | 3.55 | -102 | -49 | -5 |
| TLCA-3S | 562.4 | 482.4 | 3.55 | -97 | -50 | -5 |
| TLCA-3S_q2 | 562.4 | 562.3 | 3.55 | -90 | -11 | -5 |
| TCA | 514.3 | 80.0 | 3.57 | -200 | -123 | -5 |
| TCA_q2 | 514.3 | 514.3 | 3.57 | -140 | -30 | -5 |
| GCDCA | 448.3 | 74.0 | 4.49 | -120 | -66 | -11 |
| GCDCA_q2 | 448.3 | 448.3 | 4.49 | -108 | -20 | -5 |
| TCDCA_q2 | 498.3 | 80.0 | 4.50 | -180 | -118 | -5 |
| TCDCA | 498.3 | 124.0 | 4.50 | -180 | -74 | -10 |
| TDCA | 498.4 | 123.8 | 4.75 | -182 | -74 | -5 |
| TDCA_q2 | 498.4 | 80.0 | 4.75 | -175 | -70 | -5 |
| GDCA | 448.4 | 74.0 | 4.82 | -105 | -78 | -5 |
| GDCA_q2 | 448.4 | 448.4 | 4.82 | -110 | -17 | -5 |
| UDCA | 391.3 | 391.3 | 5.31 | -120 | -10 | -5 |
| CA | 408.3 | 289.9 | 5.48 | -140 | -50 | -5 |
| CA_q2 | 408.3 | 343.2 | 5.48 | -124 | -46 | -5 |
| TLCA | 482.4 | 80.0 | 6.46 | -166 | -112 | -5 |
| TLCA_q2 | 482.4 | 124.0 | 6.46 | -171 | -70 | -5 |
| GLCA | 432.4 | 74.0 | 6.47 | -132 | -70 | -10 |
| GLCA_q2 | 432.4 | 388.2 | 6.47 | -128 | -45 | -10 |
| CDCA | 391.6 | 391.3 | 8.00 | -170 | -16 | -5 |
| CDCA_q2 | 391.3 | 373.3 | 8.00 | -136 | -46 | -5 |
| DCA | 391.4 | 391.4 | 8.37 | -125 | -11 | -5 |
| DCA_q2 | 391.4 | 345.2 | 8.37 | -110 | -47 | -5 |
| LCA | 375 | 375.0 | 10.90 | -137 | -11 | -5 |
| LCA_q2 | 421.3 | 375.5 | 10.90 | -50 | -20 | -4 |
