# Supplementary material for: Sarcopenia: investigation of metabolic changes and its associated mechanisms
Source: Skelet Muscle. 2023 Jan 19;13:2. doi: 10.1186/s13395-022-00312-w (PMC9850598; doi:10.1186/s13395-022-00312-w)
Supplement: Supplementary file 3 — Additional file 3: Supplementary Table 2. Result from Quantitative Enrichment Analysis. [file 13395_2022_312_MOESM3_ESM.docx]

**Supplementary Table 2.** Result from Quantitative Enrichment Analysis

| **Metabolite set** | **Total Compounds** | **Hits** | **Statistic Q** | **Q Expected** | | **Q Raw** | **p Holm** | **p FDR** |
| --- | --- | --- | --- | --- | --- | --- | --- | --- |
| CARNITINE PALMITOYL  TRANSFERASE DEFICIENCY (II) | 8 | 4 | 33.11 | 4.76 | 4.62E-04 | | 5.26E-02 | 5.26E-02 |
| LONG-CHAIN-3-  HYDROXYACYL-COA DEHYDROGENASE DEFICIENCY  (LCHAD) | 10 | 3 | 18.23 | 4.76 | | 3.95E-03 | 4.46E-01 | 1.89E-01 |
| CARNITINE PALMITOYL  TRANSFERASE DEFICIENCY (I) | 5 | 2 | 21.82 | 4.76 | | 4.97E-03 | 5.57E-01 | 1.89E-01 |
| VERY-LONG-CHAIN ACYL  COA DEHYDROGENASE  DEFICIENCY (VLCAD) | 21 | 11 | 12.49 | 4.76 | | 8.38E-03 | 9.30E-01 | 1.93E-01 |
| PEARSON SYNDROM | 3 | 1 | 29.88 | 4.76 | | 8.48E-03 | 9.33E-01 | 1.93E-01 |
