# Supplementary figures and images for: Sarcopenia: investigation of metabolic changes and its associated mechanisms
Source: Skelet Muscle. 2023 Jan 19;13:2. doi: 10.1186/s13395-022-00312-w (PMC9850598; doi:10.1186/s13395-022-00312-w)

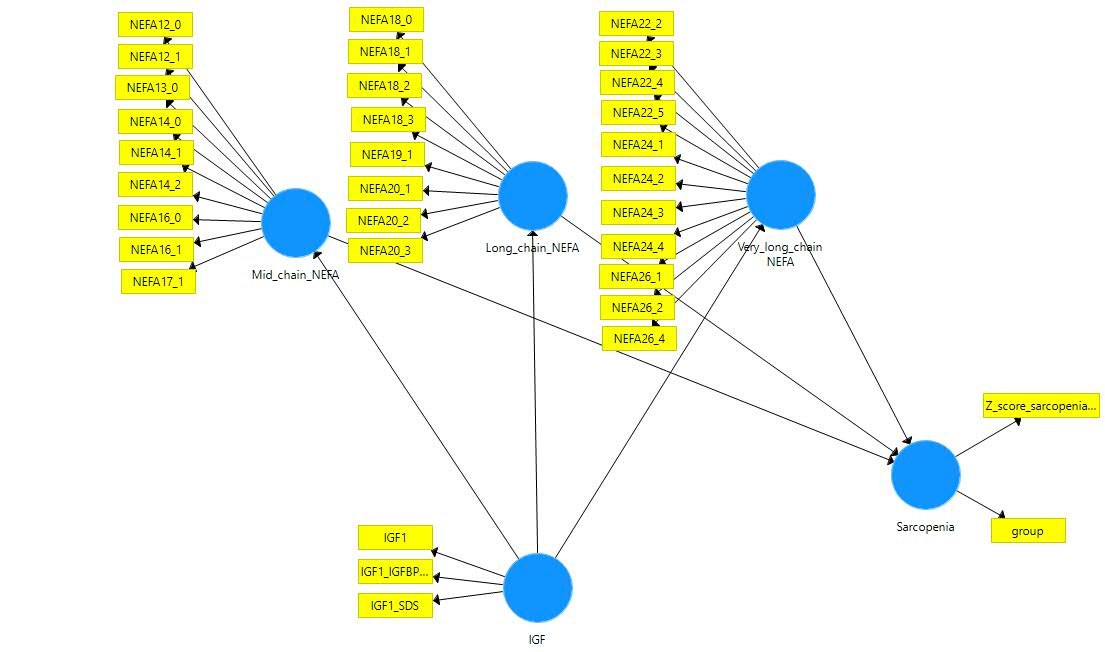

Supplement: Supplementary file 5 — Additional file 5: Supplementary Figure 1. Path model by PLSSEM showing causal effect relationship from IGF-I to sarcopenia mediated by NEFA. [file 13395_2022_312_MOESM5_ESM.jpg]
